# The Peaceful Co-existence of Input Frequency and Structural Intervention Effects on the Comprehension of Complex Sentences in German-Speaking Children

**DOI:** 10.3389/fpsyg.2017.01590

**Published:** 2017-09-29

**Authors:** Flavia Adani, Maja Stegenwallner-Schütz, Talea Niesel

**Affiliations:** Department of Linguistics, University of Potsdam, Potsdam, Germany

**Keywords:** relative clauses, sentence comprehension, input frequency, number, animacy, language acquisition, German

## Abstract

The predictions of two contrasting approaches to the acquisition of transitive relative clauses were tested within the same groups of German-speaking participants aged from 3 to 5 years old. The input frequency approach predicts that object relative clauses with inanimate heads (e.g., *the pullover that the man is scratching*) are comprehended earlier and more accurately than those with an animate head (e.g., *the man that the boy is scratching*). In contrast, the structural intervention approach predicts that object relative clauses with two full NP arguments mismatching in number (e.g., *the man that the boys are scratching*) are comprehended earlier and more accurately than those with number-matching NPs (e.g., *the man that the boy is scratching*). These approaches were tested in two steps. First, we ran a corpus analysis to ensure that object relative clauses with number-mismatching NPs are not more frequent than object relative clauses with number-matching NPs in child directed speech. Next, the comprehension of these structures was tested experimentally in 3-, 4-, and 5-year-olds respectively by means of a color naming task. By comparing the predictions of the two approaches within the same participant groups, we were able to uncover that the effects predicted by the input frequency and by the structural intervention approaches co-exist and that they both influence the performance of children on transitive relative clauses, but in a manner that is modulated by age. These results reveal a sensitivity to animacy mismatch already being demonstrated by 3-year-olds and show that animacy is initially deployed more reliably than number to interpret relative clauses correctly. In all age groups, the animacy mismatch appears to explain the performance of children, thus, showing that the comprehension of frequent object relative clauses is enhanced compared to the other conditions. Starting with 4-year-olds but especially in 5-year-olds, the number mismatch supported comprehension—a facilitation that is unlikely to be driven by input frequency. Once children fine-tune their sensitivity to verb agreement information around the age of four, they are also able to deploy number marking to overcome the intervention effects. This study highlights the importance of testing experimentally contrasting theoretical approaches in order to characterize the multifaceted, developmental nature of language acquisition.

## Introduction

Child language acquisition is a multifaceted process, which is likely to be influenced by several factors including structural rule learning, statistical learning, and social learning (e.g., Gervain and Mehler, 2010). The performance of children in experimental studies on complex sentences has often been used as a prism to infer which factors can be deployed to achieve an adult-like interpretation (e.g., Roeper, 2007). Among complex sentences, relative clauses have been used to test different language acquisition theories (Ambridge and Lieven, 2011).

The study presented in this paper was specifically designed to test, within the same participant groups, the predictions of what we will be calling the *input frequency* approach and the *structural intervention* approach. While the input frequency approach mainly concentrates on the distributional factors that influence children's early linguistic knowledge and its usage, the structural intervention approach focuses on grammatical mechanisms that may hinder or enhance the emergence of this knowledge. So far, the predictions of these approaches have been only tested in separate studies, using different participant groups and methods. Thus, the potential co-occurrence of frequency- and structure-driven effects, which might, simultaneously or successively, guide the performance of children during development can only be inferred indirectly.

In our study, we address the question of which factors support the comprehension of relative clauses by children from a new angle. First, a corpus study was conducted to identify whether the predictions of the structural intervention account, with respect to number dissimilarity effects, differ from the predictions of the input frequency account, regarding the frequency of number dissimilarities in the input. Next, we used a novel experimental design to draw a direct comparison of the predictions of the input frequency and structural intervention approaches, within the same participants and across different age groups, namely 3-, 4-, and 5-year-olds. To anticipate our findings, we were able to uncover that the effects predicted by input frequency and by the structural intervention approaches co-exist and that they both influence children's performance on transitive relative clauses, but in a way that is modulated by age. These effects hold at the group level but they are also reflected at the level of participant's individual performance.

The paper is organized as follows: First, the rationales behind the input frequency and the structural intervention approaches will be introduced and the existing studies on animacy and number dissimilarity (the two factors that are manipulated in our experiment) will be reviewed. Next, the hypotheses made by the two theoretical approaches will be tested by means of a corpus study and an experimental study. A discussion of these results and of the co-existence of frequency and structural factors on the development of complex sentences will conclude the paper.

### The input frequency approach

At the core of the input frequency approach lies the question of which environmental factors influence the emergence of children's early linguistic knowledge and its usage (Tomasello, 2000; Lieven, 2010). A few published studies have addressed this question with regard to the acquisition of relative clauses.

Based on the analysis of spontaneous speech data, Diessel and Tomasello (2000) proposed that, up to 3 years of age, English-speaking children's mastery of relative clauses is limited to structures that occur frequently in their own repertoires and which have a simple communicative function e.g., presentational constructions such as *Here's a tiger that's gonna scare him*. These sentences were analyzed as expressing a single proposition i.e., the tiger is going to scare him (cf. also Brandt et al., 2008 for converging evidence in the acquisition of German). At the same time, children's production of unequivocally fully-fledged relative clauses embedded in a main clause was mostly limited to subject-extracted relatives with intransitive verbs [e.g., *Is this something that turn(s) around?*]. Diessel and Tomasello (2000) showed that children begin to produce fully transitive subject and object relative clauses mostly between 4 and 5 years of age. The ability to correctly repeat mostly relative clauses with intransitive verbs was also found in English- and German-speaking 4-year-olds (Diessel and Tomasello, 2005), while the repetition of transitive relative clauses was significantly less accurate. Next, Kidd et al. (2007) designed a sentence repetition task where the properties of the sentences to be repeated reflected the distributional frequencies of these constructions in the input. The analysis of adult speech corpora showed that subject-extracted relative clauses tend to be more frequent than object-extracted relative clauses (Roland et al., 2007). Moreover, object relatives typically occur with an inanimate head noun and/or with a pronoun as embedded subject (e.g., *the car that she borrowed had a low tyre*) rather than with two animate NPs as verb arguments (e.g., *the cat that the dog is chasing is running very fast*). This pattern appeared to be fairly robust across (also typologically different) languages, such as English (Fox and Thompson, 1990), German (Mak et al., 2002), and Hebrew (Arnon, 2010)^1^. A number of adult sentence processing studies pointed toward a facilitation in object relative clauses with inanimate head nouns (Traxler et al., 2002; Gennari and MacDonald, 2009; Wells et al., 2009) and with embedded pronominal subjects (Reali and Christiansen, 2007), compared to object relative clauses with two animate full NPs. Based on these findings, Kidd et al. (2007) put forward the hypothesis that, if children's language processing system obeys the same constraints as the adult system, children should be able to repeat more faithfully object relative clauses with inanimate head nouns and embedded pronominal subjects. Indeed, this is what was found in English- and German-speaking 3- and 4-year-olds' productions. In English, the proportion of correctly repeated object relative clauses with an inanimate head and an embedded pronominal subject was similar to the proportion of correctly repeated subject relative clauses (~60% correct). In German, the proportion of correctly repeated, most frequently attested object relative clause structure was even higher than correctly repeated subject relative clauses. This discrepancy, however, seems to be rather due to the subject relatives, whose accuracy was surprisingly low (~20%) compared to object relatives (~60% correct). Similar accuracy patterns were obtained for comprehension by Brandt et al. (2009) by testing English- and German-speaking 3-year-olds using a referent selection task. In both languages, object relative clauses with two animate NPs were less accurate than object relative clauses with inanimate heads, which were, in turn, at least as accurate as subject relative clauses^2^. Independently of the work conducted within the input frequency approach, animacy effects during relative clause comprehension were also investigated in French-speaking 5- to 11-year-olds (Bentea et al., 2016) and Italian-speaking 9-year-olds (Arosio et al., 2011), showing converging results to the ones outlined above with younger children.

### The structural intervention approach

Differently from the input frequency approach, the structural intervention approach aims at identifying which grammatical mechanisms may hinder or enhance the emergence of children's early linguistic knowledge (Guasti, 2002; Hyams and Orfitelli, 2017). With respect to the acquisition of relative clauses, Friedmann et al. (2009) argued that it is the structural similarity between the embedded subject and the head noun that hinders the comprehension of object relative clauses with two animate NPs, such as *Show me the cat that the dog is chasing*. The two NPs are structurally similar in the sense that they both contain an overt noun (*cat, dog*), a “lexical restriction” as Friedmann et al. called it. This overt noun on the embedded subject NP (*dog*) intervenes between the head noun (*cat*) and the position where this noun is interpreted as an object of the verb *chase*. This position and the one where the noun is pronounced as relative clause head are argued to be connected via syntactic movement. According to Friedmann et al. (2009), but cf. also Grillo (2009), the structurally similar, intervening subject NP disrupts the establishment of the movement dependency and the correct interpretation of the sentence. Friedmann et al. (2009) tested Hebrew-speaking 3- to 5-year-olds (mean age 4;6) by comparing the production and comprehension of object relative clauses with two animate full NPs with those where only one argument is a full NP, such as object free relatives e.g., *Show me the who that the dog is chasing*, a well-formed sentence of Hebrew (as well as other conditions). Children were more accurate on those conditions where only one of the two verb arguments contained an overt noun (e.g., object free relatives), compared to object relatives with two full NPs. Hence, the prediction that the presence of an overt noun in the full NP hinders the comprehension of object relative clauses was borne out. By testing Italian- and English-speaking children, Adani et al. (2010, 2014) refined the notion of structural similarity taking into account grammatical features that are encoded within the NP, such as gender and number. As for English, Adani et al. (2014) showed that center-embedded subject as well as object relative clauses where the embedded NP and the head NP differ in terms of number (i.e., one is plural and the other is singular as in *The cat that is washing the goats/that the goats are washing has climbed onto the stool*) were understood significantly more accurately than the same structures without number dissimilarity (i.e., where both nouns are singular as in *The cat that is washing the goat/that the goat is washing has climbed onto the stool*). This result suggests that NP-internal features, like number, are relevant in the computation of structural intervention. Moreover, Adani et al. (2010) tested Italian center-embedded object relative clauses similar to the English study in three groups of Italian-speaking 5-, 7-, and 9-year-olds. The number dissimilarity effect was replicated, but Italian added the possibility to test also gender-marking. In contrast to number, gender dissimilarities yielded a significantly smaller facilitation effect. Hence, Adani et al. (2010) conclude that not all NP-internal features are equally relevant in the computation of structural intervention effects. The reduced facilitation of gender marking in the facilitation of object relative clauses in Italian was also tested in a subsequent study by Belletti et al. (2012), where Italian and Hebrew were compared. Subject- and object relative clauses of the type *Show me the dog that the goat is chasing* were tested separately in Hebrew and Italian in two groups of 3- to 5-year-olds (mean age 4;7 for each language). Italian and Hebrew crucially differ with respect to gender-marking: in Italian, it is only marked on nouns (e.g., dog is masculine and goat is feminine) while in Hebrew it is marked on the noun as well as on the verb via subject-verb agreement. Belletti et al. (2012) found that gender marking facilitates the comprehension of relative clauses in Hebrew but not (or, rather, to a much lesser extent) in Italian, similarly to what Adani et al. (2010) also found. Hence, Belletti et al. (2012) argued that a facilitation in structural intervention configurations only comes from features that are triggers of syntactic movement, typically inflected on the verb (e.g., number in English and in Italian, gender in Hebrew).

The hypothesis put forward by Belletti et al. (2012) is precisely the version of the structural intervention account that we will investigate in the present study and whose predictions will be compared to the predictions of the input frequency account (see Riches and Garraffa, 2017 for pursuing a similar goal but focusing on different structures). The properties of German suit well these purposes as we know from previous studies that object relative clauses with inanimate heads are more frequent in the input (Mak et al., 2002), easier to imitate (Kidd et al., 2007) and to comprehend (Brandt et al., 2009) than object relative clauses with two animate NPs. Moreover, similarly to English and Italian, number agreement is overtly marked on verbs in German (Eisenberg, 2013). Coming to the predictions for our study, the input frequency approach predicts object relative clauses with an inanimate head and an animate embedded subject (OR:IN-AN, 1) to be more accurate than object relative clauses with two animate and singular NPs (OR:AN-AN, 2). The structural intervention approach predicts OR:AN-AN to be harder than object relative clauses with a singular head and a plural embedded NP (OR:SG-PL, 3)^3^.

1. The pullover that the man is scratching (OR:IN-AN)
2. The man that the boy is scratching (OR:AN-AN)
3. The man that the boys are scratching (OR:SG-PL)

In order to address further predictions of the two approaches, two types of subject relative clauses were tested as well. Based on the previous studies conducted in the spirit of the input frequency approach, OR:IN-AN are expected to be as accurate (or even more accurate) than subject relative clauses with two animate NPs (SR:AN-AN, 4). On the other hand, the structure intervention approach predicts the number marking facilitation to be specific for object relative clauses. Hence, when comparing SR:AN-AN with subject relative clauses with a singular head and a plural embedded NP (SR:SG-PL, 5), the structural intervention account predicts no difference between the two:

4. The man that is scratching the boy (SR:AN-AN)
5. The man that is scratching the boys (SR:SG-PL)

Crucially, however, it is not clear from the published literature whether a number facilitation in these contexts could also be predicted by the input frequency approach. According to the structural intervention account, object relative clauses with one singular NP and one plural NP are expected to be easier to interpret than object relative clauses with two verb arguments of the same number. Number is a movement-triggering feature and helps to reduce intervention between the moved head NP (the object) and the embedded NP (the subject). A facilitation in the same direction would be predicted by the input frequency account only if object relative clauses with one singular NP and one plural NP were more frequent in the input than object relative clauses with two verb arguments of the same number. To our knowledge, the question of how frequent relative clauses with number dissimilarity are (compared to relative clauses with number match), has not been examined yet in the existing literature. In order to set the basis for our experimental study, we report the data of a corpus study in which this question was addressed.

## Materials and methods

### Corpus study

In order to examine the input frequency of relative clauses with and without number mismatch between the head NP and the embedded NP, the speech of adults interacting with three German-speaking children from the CHILDES corpus (MacWhinney, 2000) was analyzed. The three corpora were those of Caroline (age range: 0;1–4;3), Kerstin (1;3–3;4), and Simone (1;9–4;0). All relative clauses containing the relative pronouns *der, die, das, den, wer, was, welcher, welches, welche*, and *wo* were extracted for a total of 307 utterances. All sentences were coded by the first author and subsequently checked by a native speaker with a linguistic background who was blind to the purpose of the analysis. All utterances were first classified as subject (SR, *N* = 134) or object (OR, *N* = 173) relative clauses. Among subject relative clauses, the ones containing the copular verb *sein* (to be) (*N* = 38, 28.5%), an intransitive verb (*N* = 59, 44%), or a reflexive verb (*N* = 5, 3.7%) were excluded from further analysis, leaving us with a total of 32 transitive subject relative clauses. For all transitive relative clauses, we analyzed whether the two NPs displayed (a) the same number; (b) a different number. Moreover, the two NPs were further analyzed in terms of their animacy properties, whether they are: (c) both animate; (d) the head noun is inanimate and the embedded NP is animate; (e) the head noun is animate and the embedded NP is inanimate; (f) both inanimate. These results are summarized in Table 1.

**Table 1:** Distribution of subject- and object- NP types in German child-directed speech.

**SR (*****N*** **= 32)**				**OR (*****N*** **= 173)**			
**(a) Number match**		**(b) Number mismatch**		**(a) Number match**		**(b) Number mismatch**	
**24**		**8**		**115**		**58**	
**(c) AN-AN**	**(d) IN-AN**	**(e) AN-IN**	**(f) IN-AN**	**(c) AN-AN**	**(d) IN-AN**	**(e) AN-IN**	**(f) IN-IN**
6	1	19	6	21	141	3	7

*SR, subject relative clauses; OR, object relative clauses; Number match, both NPs are singular or plural; Number Mismatch, one NP is singular and one is plural; AN-AN, both NP are animate; IN-AN, head NP is inanimate and embedded NP is animate; AN-IN, head NP is animate and embedded NP is inanimate; IN-IN, both NPs are inanimate*.

The distribution of NP types in object relative clauses was analyzed statistically with respect to two relevant comparisons: the occurrence of inanimate head nouns and the occurrence of number mismatch. Object relative clauses with inanimate heads were more frequent than object relative clauses with two animate NPs (binomial test, *p* < 0.001) and both subject- and object relative clauses with number mismatch are rarer than their number match counterparts (binomial test for subject relatives, *p* = 0.007; for object relatives, *p* < 0.001).

To recap, some familiar and novel patterns emerge from this corpus study. First, over 70% of the subject relatives occurring in the child directed speech were either containing a copular verb or an intransitive verb. Although our analyses focused on transitive relative clauses only, the frequent occurrence of presumably simpler structures is noteworthy given previous claims that children's mastery of relative clauses before 3 years of age is limited to structures that occur frequently in the input, namely copular and intransitive relative clauses (Diessel and Tomasello, 2000, 2005). Second, both subject and object relative clauses with two animate NPs are rare in child directed speech and, within object relative clauses, significantly less frequent than object relative clauses with an inanimate head and an animate embedded subject, as also argued previously (Kidd et al., 2007; Brandt et al., 2009). We also found that object relative clauses with an animate head and an inanimate embedded subject are, overall, extremely rare and so are object relative clauses with two inanimate arguments. Most importantly, the novel information that emerges from this corpus study is that subject- and object relative clauses where the two NPs have different number are significantly rarer than the same structures where the two NPs display the same number. We can therefore conclude that, if a facilitation of object relative clauses with number dissimilarity will be attested in the experimental task, this is unlikely to be explicable on the basis of input frequency.

Coming to the experimental study, we put forward to following predictions, where “>” means “more accurate than” and “ = ” means “as accurate than as”:

- Under the input frequency approach, OR:IN-AN > OR:AN-AN. Moreover, many studies testing this approach found that OR:IN-AN = SR:AN-AN;
- Under the structural intervention approach, OR:SG-PL > OR:AN-AN. A derived but related prediction is that SR:AN-AN = SR:SG-PL;
- The developmental trajectory will inform us whether the effects predicted by input frequency and structural intervention approaches co-exist or not;

### Experimental task

#### Participants

Seventy-three monolingual German-speaking children participated and were divided into three age groups: 23 three-year-olds (mean age 3;7, range 3;1–3;11), 25 four-year-olds (mean age 4;6, range 4;0–4;11), and 25 five-year-olds (mean age 5;4, range 5;0–5;11). Six additional children were tested but later excluded for one following reasons: lack of task completion (*N* = 1), difficulty in distinguishing the depicted characters (*N* = 1), color blindness, as indicated in the parental questionnaire (*N* = 1), failure to name three colors during the pre-test (*N* = 1), history of speech therapy (*N* = 2). This study was reviewed and approved by ethic committee at the University of Potsdam and it was carried out with parental written informed consent from all participants, in accordance with the Declaration of Helsinki. The study was piloted with a group of 4- to 5-year-olds and a group of adults (Adani, 2012) and later slightly modified in order to address the new research questions put forward in this paper. All children reported in this paper were tested with this updated version of the material.

#### Material

The test sentences were transitive object- (OR, 6–8) and subject (SR, 9–10) relative clauses, for a total of 20 trials (four items per condition). Differently from English, both subject and object relative clauses display the finite verb in clause final position in German, thus creating minimal pairs between the two extraction types, which are not confounded by overt word order differences. In addition to the test sentences, 16 relative clauses with intransitive verbs, e.g., (11), and prepositional phrases, e.g., (12), were included as fillers, for a total of 36 trials per list.

6. Welche Farbe hat der Mann, den die Jungen kratzen? (OR:SG-PL)
  Which color has the man whom the boys scratch
7. Welche Farbe hat der Mann, den der Junge kratzt? (OR:AN-AN)
  Which color has the man whom the boy scratches
8. Welche Farbe hat der Pulli, den der Mann kratzt? (OR:IN-AN)
  Which color has the pullover whom the man scratches
9. Welche Farbe hat der Mann, der den Jungen kratzt? (SR:AN-AN)
  Which color has the man who scratches the boy?
10. Welche Farbe hat der Mann, der die Jungen kratzt? (SR:SG-PL)
  Which color has the man who scratches the boys?
11. Welche Farbe hat der Mann, der trinkt? (Filler, intransitive)
  Which color has the man who drinks?
12. Welche Farbe hat der Pulli an der Wäscheleine? (Filler, prepositional)
  Which color has the pullover on the clothesline?

**Figure 1:**
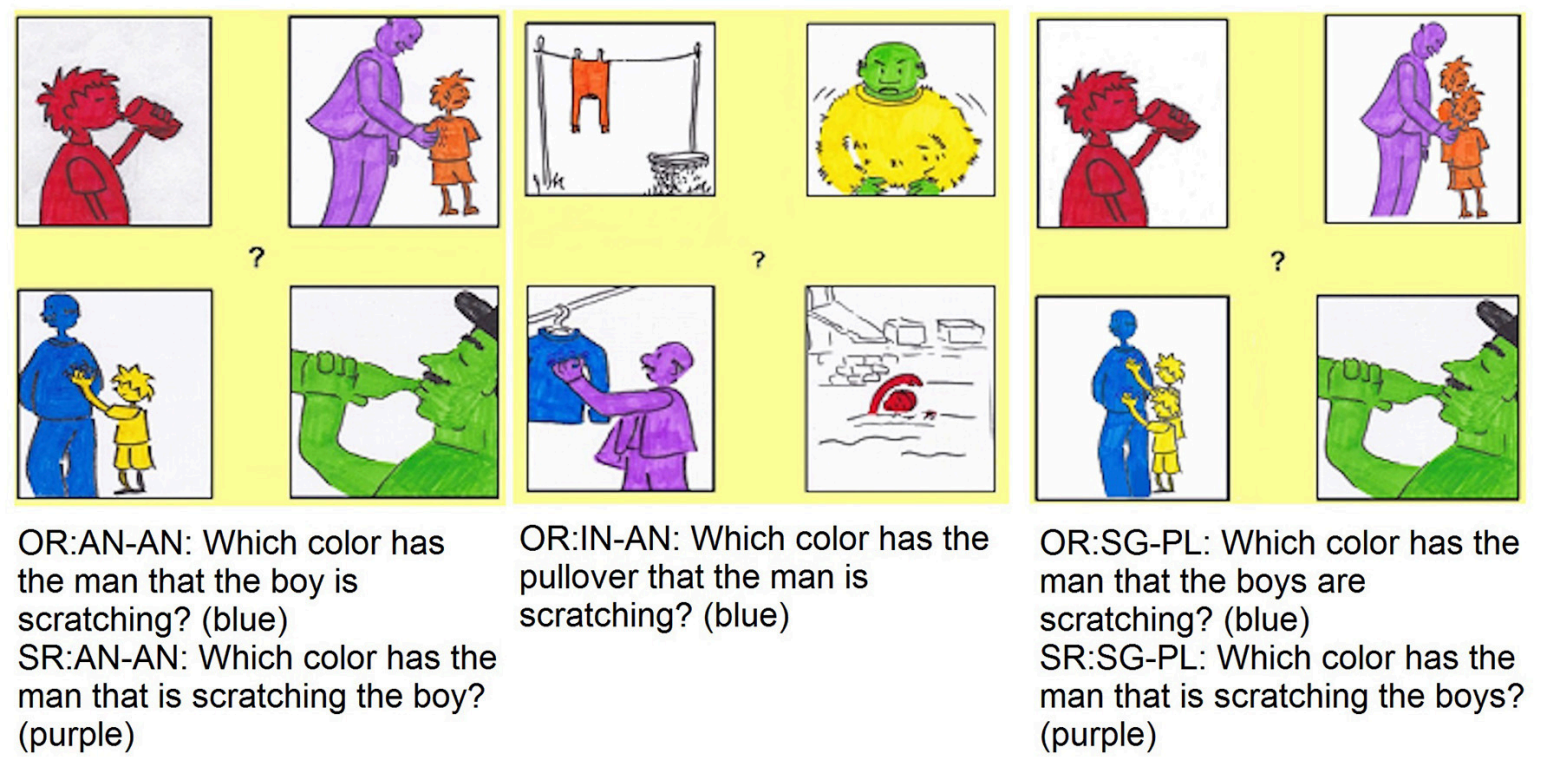
Example of the visual stimuli, related test sentences (English translation) and expected answer (in brackets). OR, object relative clause; SR, subject relative clause; AN-AN, two animate, singular NPs; IN-AN, one inanimate NP and one animate NP, both singular; SG-PL, one singular NP and one plural NP, both animate.

All trials were pseudo-randomized, with one filler after maximally three test sentences and it was never the case that two relative clauses of the same type followed each other. To neutralize order effects, the stimuli were administered in one order to half of the participants and in the reversed order to the other half. One stimulus list is reported in the Supplementary Material. Figure 1 provides an example of three visual displays used for testing each of the five conditions.

Each visual display contained four pictures with characters of different colors. Only one picture displayed the target referent, e.g., a pullover in (8 and 12) or a man in the other conditions, performing the action expressed by the verb, e.g., scratching, and assuming the correct thematic role (either the agent or the patient). Each four-picture configuration was used to test one subject- and one object relative clause as well as two filler sentences, except in the animacy contrast, where subject relative clauses were not tested. Five additional characters in the scene were coded according to five non-target responses. In this paper, we decided not to report an analysis of non-target responses but the interested reader can find an overview in the Supplementary Material.

#### Procedure

The testing session started with a pre-test to ensure that children were familiar with the nouns: *Junge* (boy), *Baumstamm* (log), *Mann* (man), *Eimer* (bucket), *Gurt* (belt), *Schuh* (shoe), *Pulli* (pullover), *Frau* (lady), the verbs: *drücken* (to squeeze, to hug), *halten* (to carry, to hold), *kratzen* (to scratch), *tragen* (to carry, to hold) and the colors: *grün* (green), *lila* (purple), *blau* (blue), *orange* (orange), *gelb* (yellow), *rot* (red) used in the experiment. The lexical items, and in particular, the verbs, were chosen in such a way that either an animate or an inanimate noun could be a plausible subject. The interaction between the experimenter and the child was mediated by a snail puppet named Bala who introduced the sentence-picture-matching task in the form of a color naming game, inspired by Arnon (2010). The precise task instructions are reported in the Supplementary Material. At most one repetition of the trial was allowed. Before the experiment started, the participant was familiarized to the task by means of four practice trials. In addition to the color naming task, we administered a standardized non-word repetition task (Grimm et al., 2010), a selective attention test (Grob et al., 2009), a phonological memory test (Grob et al., 2009), and a sentence comprehension test (Siegmüller et al., 2011). These tests were used as background measure for another study which is not reported in this paper. Each participant was tested individually by means of one or two sessions (depending on the child's individual pace and motivation) either in a quiet room of the day care or in a university laboratory. The whole testing lasted about 50 min for each participant, with breaks when needed. Children were generally engaged and happy to participate, and received a small toy as a reward.

## Results

**Figure 2:**
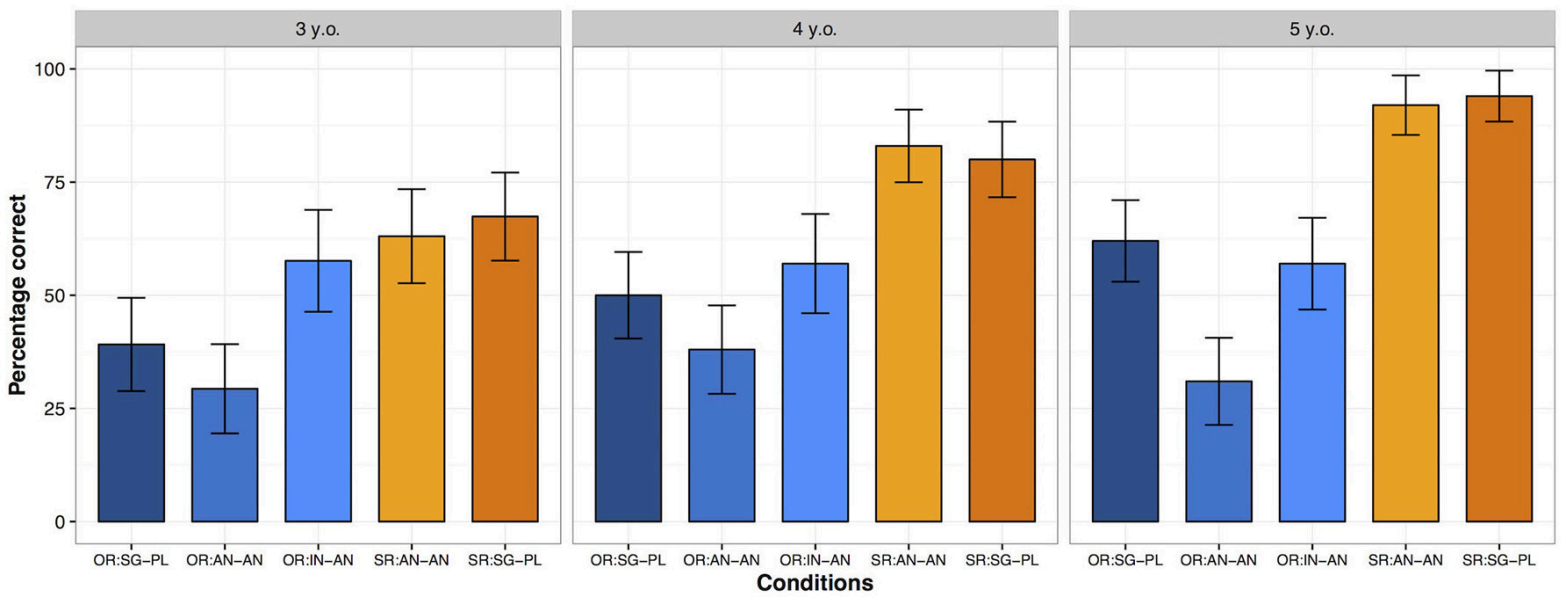
Distribution of correct responses (in percentages) in each experimental condition, across age groups. The error bars represent 2 standard errors of the mean. OR, object relative clause; SR, subject relative clause; AN-AN, two animate and singular NPs; IN-AN, one inanimate NP and one animate NP, both singular; SG-PL, one singular NP and one plural NP, both animate.

All responses were scored according to their correctness to the color naming question. Percentages of correct responses in filler sentences as well as test sentences were computed. Filler sentences were generally above 90% accurate, with some variation among 3-year-olds (92%), 4-year-olds (97%), and 5-year-olds (99%). Percentage of correct responses in each experimental condition is illustrated in Figure 2.

Visual inspection of Figure 2 reveals that, as expected, subject relative clauses are overall more accurate than object relative clauses, when all conditions are taken together. The accuracy of object relative clauses varies considerably across conditions and age groups. The following statistical analysis was conducted to gain more insight as to which differences are statistically different across age groups.

We analyzed the proportion of correct responses with generalized linear mixed-models within the lme4 package (Bates et al., 2015b) in R (version 3.2.2; R Core Team, 2015). A logistic link function was used because of the binary dependent variable which could either have the value 1 (correct) or 0 (incorrect). We will present a statistical model that contains *group* and *condition* as categorical variable in its fixed effects structure. The three levels of group were contrasted in two steps: first, 4 year-olds (coded as 1) vs. 3 year-olds (coded as −1); next, 5 year-olds (coded as 1) vs. 4 year-olds (coded as −1). To address our research questions, we planned the following condition comparisons (the contrast coding is indicated in brackets):

f1: OR:AN-AN (1) vs. OR:SG-PL (−1) −> Is there a number dissimilarity facilitation?
f2: OR:IN-AN (1) vs. OR:AN-AN (−1) −> Is there an animacy dissimilarity facilitation?
f3: SR:AN-AN (1) vs. OR:IN-AN (−1) −> Are OR with an inanimate head as easy as SR?
f4: SR:SG-PL (1) vs. SR:AN-AN (−1) −> Is the number dissimilarity effect present also in SR?

The model tested for main effects of group and specific condition comparisons as well as interactions between group x condition. Following Bates et al. (2015a), we run a principle component analysis to identify the maximal model with only those random effects components that are supported by the data. The goodness-of-fit of nested alternatives of this model's random effects structure were evaluated with the *anova* function in R, based on the *p*-value associated to the chi-square-distributed likelihood ratio (Matuschek et al., 2017). After these checks, the random effects structure of the final model included varying subject intercepts and slopes for the comparison OR:IN-AN/OR:AN-AN and SR:AN-AN/OR:IN-AN as well as their correlation parameters. To sustain model convergence, we specified the *bobyqa* optimizer in the *glmer* function. The final model is the following:

$$\begin{array}{l} m<-glmer(accuracy\sim group+f1+f2+f3+f4+group:\\ \;f1\;+\;group:f2\;+\;group:f3\;+\;group:f4\;+\;(1\;+\;f2\\ \;+\;f3\;|\;subject\_id),\;family=binomial,\;control\\ \;=glmerControl(optimizer=“bobyqa”),data)) \end{array}$$

The output of this model is reported in Table 2. The statistically significant effects are highlighted in gray:

**Table 2:** Model output.

**Fixed effects**	**Estimate**	**SE**	***z*-value**	***p*-value**
(Intercept)	0.643	0.134	4.789	<0.001
4 y.o. vs. 3 y.o.	0.637	0.309	2.062	0.039
5 y.o. vs. 4 y.o.	0.532	0.320	1.663	0.096
OR:AN-AN vs. OR:SG-PL	−1.007	0.208	−4.851	<0.001
OR:IN-AN vs. OR:AN-AN	1.277	0.237	5.393	<0.001
SR:AN-AN vs. OR:IN-AN	1.547	0.257	6.013	<0.001
SR:SG-PL vs. SR:AN-AN	0.113	0.262	0.430	0.667
4 vs. 3 y.o. x OR:AN-AN vs. OR:SG-PL	−0.110	0.492	−0.222	0.824
5 vs. 4 y.o. x OR:AN-AN vs. OR:SG-PL	−1.105	0.497	−2.222	0.026
4 vs. 3 y.o. x OR:IN-AN vs. OR:AN-AN	−0.487	0.572	−0.850	0.395
5 vs. 4 y.o. x OR:IN-AN vs. OR:AN-AN	0.472	0.562	0.838	0.402
4 vs. 3 y.o. x SR:AN-AN vs. OR:IN-AN	1.348	0.546	2.469	0.014
5 vs. 4 y.o. x SR:AN-AN vs. OR:IN-AN	0.968	0.624	1.551	0.121
4 vs. 3 y.o. x SR:SG-PL vs. SR:AN-AN	−0.480	0.530	−0.905	0.365
5 vs. 4 y.o. x SR:SG-PL vs. SR:AN-AN	0.572	0.705	0.811	0.418

*OR, object relative clause; SR, subject relative clause; AN-AN, two animate and singular NPs; IN-AN, one inanimate NP and one animate NP, both singular; SG-PL, one singular NP and one plural NP, both animate*.

For interactions that were statistically significant in the main model, we nested the pairwise comparisons in order to explain the directions of the interactions. The significant pairwise comparisons are reported in the text below. The complete output of the two models with nested effects for the significant interactions are reported in the Supplementary Material.

We found significant main effects of group and of three out of the four pre-planned comparisons. The significant main effect of group reveals that 3-year-olds perform significantly less accurately (*M* = 51%) than 4-year-olds (*M* = 62%), but the difference between 4- and 5-year-olds (*M* = 67%) does not reach significance.

The effect of the condition comparison OR:AN-AN vs. OR:SG-PL reveals that object relative clauses with number dissimilarity are more accurate (*M* = 51%) than object relative clauses with two animate, singular NPs (*M* = 33%). A significant interaction between group and OR:AN-AN vs. OR:SG-PL and subsequent pairwise comparisons reveal that, for 4- and especially for 5-year-olds, object relative clauses with number dissimilarity are significantly more accurate than object relative clauses with two animate, singular NPs (4 year-olds: β = −0.675, SE = 0.340, z = −1.987, *p* = 0.047; 5 year-olds: β = −1.780, SE = 0.369, z = −4.824, *p* < 0.001). In 3-year-olds, this effect shows a similar direction but it is not significant (β = −0.566, SE = 0.358, z = −1.581, *p* = 0.114). Moreover, the effect of condition comparison OR:IN-AN vs. OR:AN-AN and the absence of an interaction with group reveals that object relative clauses with an inanimate head and an animate embedded subject (*M* = 57%) are more accurate that object relative clauses with two animate NPs (*M* = 33%) and that this facilitation holds across all age groups. The effect of condition comparison SR:AN-AN vs. OR:IN-AN reveals that subject relative clauses with two animate, singular NPs (*M* = 80%) are overall more accurate than object relative clauses with an inanimate head (*M* = 57%). A significant interaction between group and SR:AN-AN vs. OR:IN-AN and subsequent pairwise comparisons reveal that subject relative clauses with two animate, singular NPs are more accurate than object relative clauses with an inanimate head for 4- and 5-year-olds (4-year-olds: β = 1.674, SE = 0.411, z = 4.074, *p* < 0.001; 5-year-olds: β = 2.642, SE = 0.500, z = 5.281, *p* < 0.001) but not for 3-year-olds (β = 0.326, SE = 0.367, z = 0.888, *p* = 0.375). There was no significant difference between subject relative clauses with two animate, singular NPs (*M* = 80%) and subject relative clauses with number dissimilarity (*M* = 81%).

### Individual performance

In order to evaluate whether these group results reflect a response behavior that also holds at the individual level, we checked how many children named the correct color on at least 3 out of 4 trials per condition, within each age group. This corresponds to a probability of *p* < 0.05 of providing the correct answer, assuming that each child has a 16.6% chance of naming the correct color. The chance level was fixed at 16.6% considering that the participant was expected to respond with one (correct) color out of six potential alternatives (100/6 = 16.6). The results of this pass/fail analysis are reported in Table 3.

**Table 3:** Number (and percentages) of children who performed accurately on at least 3 out of 4 trials per condition.

**Age group**	**OR:SG-PL (%)**	**OR:AN-AN (%)**	**OR:IN-AN (%)**	**SR:AN-AN (%)**	**SR:SG-PL (%)**
3 y.o. (*N* = 23)	5 (22)	3 (13)	11 (48)	13 (57)	15 (65)
4 y.o. (*N* = 25)	9 (36)	5 (20)	10 (40)	22 (88)	20 (80)
5 y.o. (*N* = 25)	14 (56)	3 (12)	11 (44)	24 (96)	25 (100)

The response patterns of individual participants corroborate the group results along several dimensions. Starting with subject relative clauses, we found that the majority of the 3-year-olds already performed very accurately on both types (SR:AN-AN and SR:SG-PL). This rate increases in 4-year-olds and virtually all 5-year-olds performed correctly on both types of subject relative clauses. Moving to object relative clauses, we observe that the number of children who performed accurately on object relative clauses with two animate, singular NPs (OR:AN-AN) and with an inanimate head (OR:IN-AN) does not increase as a function of age. Rather, while only a restricted subgroup (12–20%) of children succeeds on OR:AN-AN, a larger subgroup of children (40–48%) performed accurately on the condition OR:IN-AN. What is nevertheless worth to emphasize is that these rates remain fairly steady across age groups. Finally, object relative clauses with number mismatch are correctly understood only by a restricted subgroup of 3-year-olds (22%). Crucially, for the research questions addressed in this study, the relative number of children who performed correctly on the OR with number dissimilarity was low in relation to the OR with an inanimate head (5 vs. 11, respectively). But by the age of 5 years, the relation between these two conditions flips its direction. While the number of children performing accurately on the OR with inanimate heads remains fairly steady, the number of passers on OR with number dissimilarity increases (11 vs. 14, respectively). The implications of the group and individual results will be discussed in the next section.

## Discussion

A very thoroughly investigated question in the last decades' psycholinguistic research literature has been which type of information the human parser is relying on when processing filler-gap dependencies, of which relative clauses are a typical instance. Simplifying somehow a very multifaceted state-of-the-art, researchers have proposed several accounts, which capitalize on different sources of information that become crucial to achieve the correct interpretation of these sentences. For instance, some of these approaches emphasize the role of frequency (e.g., Gennari and MacDonald, 2009), some the role of memory resources (e.g., Lewis et al., 2006) and other the role of syntactic structure (e.g., De Vincenzi, 1990). Similar avenues have been pursued in the field of language acquisition. The main aim of this paper was precisely to bring together two of these approaches and test them systematically across 3-, 4-, and 5-year-olds.

The two approaches under discussion are the *input frequency* approach (Diessel and Tomasello, 2000, 2005; Kidd et al., 2007; Brandt et al., 2009; a.o) and the *structural intervention* approach (Friedmann et al., 2009; Grillo, 2009; Adani et al., 2010, 2014; Belletti et al., 2012; a.o). In order to evaluate the input frequency approach, we tested the prediction that object relative clauses with an inanimate head and an embedded animate NP are easier to interpret than object relative clauses with two animate NPs. Moreover, we also tested a derived prediction, which is often supported by the existing literature, that object relative clauses with an inanimate head are as easy as subject relative clauses with two animate NPs. On the other hand, in order to evaluate the structural intervention approach, we tested the prediction that object relative clauses with number dissimilarity are easier to interpret than object relative clauses with two singular NPs. Moreover, we have also tested the specificity of this prediction by comparing subject relative clauses with and without number dissimilarity.

In agreement with the input frequency approach, our corpus study converges with the claim that object relative clauses with an inanimate head and an embedded animate NP are the most frequent in child directed speech. Crucially, however, this analysis also revealed that object relative clauses with number dissimilarity (one singular NP and one plural NP as verb arguments) are less frequent than object relative clauses with two animate, singular NPs. This suggests that a potential facilitation in the former condition could only be explained under the structural intervention approach and not in terms of input frequency.

Our experimental data reveal that object relative clauses with an inanimate head are more accurate than object relative clauses with two animate NPs, as the input frequency approach predicts. This response pattern is attested already in 3-year-olds and the facilitation sustains developmentally, as it is also attested in 4- and 5-year-olds. In 3-year-olds, however, the animacy dissimilarity is the main factor that enhances the accuracy on object relative clauses. These results are in contrast with the claim that only presentational, mono-clausal constructions and relative clauses with intransitive verbs are mastered by 3-year-olds, as Diessel and Tomasello (2000, 2005) proposed on the basis of a corpus study. Our data support the hypothesis that 3-year-olds are able to interpret transitive fully-fledged relative clauses, as long as they are of the frequently occurring type (Brandt et al., 2009).

Despite the early occurrence and the longitudinal robustness of an animacy effect, it is noted that the accuracy level in object relative clauses with an inanimate head and an embedded animate subject does not increase as the children grow older but remains around 57–58%. This response pattern is also reflected in the analysis of individual performances where we have noticed that around half of the group of 3-year-olds is already quite accurate in this condition, by performing on at least three out of four trials correctly. However, about half of the 5-year-olds still has some difficulties in interpreting these sentences fully correctly. At this point, we can only speculate that the animacy contrast information is immediately accessible even to very young children but that the successful deployment of this information to correct sentence interpretation is based on the application of some top-down, shallow processing heuristic rather than a bottom-up, deep processing of the sentence in the adult-like sense. Based on these results, we suggest that the sensitivity to input frequency information is not subject to development, meaning that it is available from very early on but it does not increase as the child's cognitive and linguistic development progresses.

Coming to the second prediction of the input frequency approach, namely that object relative clauses with an inanimate head can become as easy as subject relative clauses, our data do not support this prediction. We have found that 4- and 5-year-olds are still significantly more accurate on subject relative clauses than on the frequent object relatives with inanimate heads. This difference is not attested in 3-year-olds, though. However, a similar performance on these two conditions in 3-year-olds has more to do with the low accuracy of subject relative clauses in the youngest group. For this reason, the difference with object relative clauses with an inanimate head fades away. This finding is similar to the one reported by Kidd et al. (2007) where 3-year-olds were only able to repeat faithfully 20% of the prompted structures. This apparent “difficulty” with subject relative clauses with two animate NPs could be linked to the lack of an animacy contrast and, as such, the impossibility for 3-year-olds to apply the above mentioned heuristic as successfully as they do with object relative clauses with inanimate heads. In the older groups, however, an advantage for subject relative clauses over object relative clauses with inanimate heads may signal that 4-year-olds start to provide a deeper, fully-fledged syntactic analysis of these structures and the interpretation of a subject relative clause does not pose a challenge. Another related observation that we leave open to future research concerns the co-occurrence of several distributional constraints that may incrementally support the comprehension of frequent object relative clauses. In this study, we have only manipulated the animacy constraint but, as noted in the introduction, the presence of an embedded pronominal subject may also play a crucial role in modulating the ease of object relative clauses. It could be that object relative clauses become as accurate as subject relative clauses only when both the animacy and the pronominal constraints are satisfied (cf. also the discussion in Arnon, 2010). We will now move to the discussion of effects related to structural intervention.

Coming to the predictions of the structural intervention approach, we found that number dissimilarity enhances the correct interpretation of object relative clauses, when all age groups are taken together. Moreover, this effect emerges in 4-year-olds but it becomes stronger in 5-year-olds. This is in line with the literature that has tested the structural intervention approach in children with a mean age of 4;6 or older ones. In all these studies, a dissimilarity of features that are triggers of syntactic movement enhanced object relative clause comprehension. These features are, for instance, number dissimilarity in Italian and English (Adani et al., 2010, 2014), gender dissimilarity in Hebrew (Belletti et al., 2012) and potentially in other languages in which subject-verb agreement is marked on the verb. There is independent evidence coming from research on subject-verb agreement using implicit measures (eye-movements) showing that between 3- and 5-years of age German-speaking children are fine-tuning their sensitivity to agreement information as well as to its violations. Brandt-Kobele and Höhle (2010) showed that 3-year-olds take advantage of the information on the verb inflection to identify the correct agreeing subject NP. However, this ability was only evident in the eye-gazes of the children but not in the explicit (pointing) responses. Considering the explicit nature of our task, in which children were asked to name the color of the relative clause head noun referent, and the fact that our test sentences are more complex that the declarative sentences tested by Brandt-Kobele and Höhle, it is not surprising that the number dissimilarity facilitation only emerges in the group of 4-year-olds. The apparent similarity between Brandt-Kobele and Höhle's data and the data of the present study suggests that the number effect might emerge already in 3-year-olds, when they are tested implicitly, for instance, measuring their eye-gazes. Nevertheless, what the explicit version of our task already shows is that, everything else being equal, there is an earlier advantage for the animacy dissimilarity over the number dissimilarity.

Coming to the second prediction of the structural intervention approach, we do find that subject relative clauses with number dissimilarity are not different from subject relative clauses without number dissimilarity. This result is in line with the predictions of the structural intervention approach, thus supporting the claim that these effects occur specifically in the intervention-triggering contexts. It may be worth to notice that the lack of statistical difference is not a consequence of an at-ceiling performance on subject relatives in general. Rather, each age group performed very similarly on the two subject relative conditions. As we have already discussed, their accuracy is quite low in 3-year-olds (SR:AN-AN: 63% correct; SR:SG-PL: 67%) and it increases gradually in 4-year-olds (SR:AN-AN: 83%; SR:SG-PL: 80%) and in the 5-year-olds (SR:AN-AN: 92%; SR:SG-PL: 94%).

The conclusions that can be drawn from our study are in agreement with most of the studies published by researchers that work with the input frequency approach and the structural intervention approach. The step forward that our study makes is to compare these two approaches directly, within the same children and across relatively large samples of participants belonging to different age groups. By doing so, we have discovered that input frequency and structural intervention effects co-exist and that the emergence of these effects is modulated by age. In all age groups, the animacy mismatch appears to explain children's performance, thus, showing that the comprehension of frequent object relative clauses is enhanced. These results reveal a sensitivity to animacy mismatch already in 3-year-olds and show that animacy is initially deployed more reliably than number to interpret relative clauses correctly. Once children fine-tune their sensitivity to verb agreement information around age four, they are also able to deploy number marking to overcome the intervention effect. Future avenues of investigation that our study opens up are the use of implicit measures, for instance eye-tracking, to shed more light on the abilities of 3-year-olds, who might not be able to cope with the explicit task demands as efficiently as the older children do. Our study also highlights the importance of comparing predictions of different developmental approaches in combination with cross-sectional data to gain detailed insight into the dynamics of the acquisition process.

## Author contributions

FA and TN conceived the study and prepared the material; FA and MS acquired and analyzed the data. FA, MS and TN drafted, revised, and approved the final manuscript.

### Conflict of interest statement

The authors declare that the research was conducted in the absence of any commercial or financial relationships that could be construed as a potential conflict of interest.

## Footnotes

^1^However, not for all these languages, the animacy and the pronoun constraints were evaluated simultaneously. Mak et al. (2002) only investigated animacy while Arnon (2010) limited her investigation to the distribution of embedded pronouns in child- and child-directed speech. Kidd et al. (2007) assessed both distributional constraints in child data.

^2^Both studies (Kidd et al. and Brandt et al.) tested, in addition to the animacy constraint, the pronoun constraint. This comparison yielded converging response patterns. But since our experiment focuses on the animacy constraint, the pronoun constraint will be not further discussed (but see also Arnon, 2010; Haendler et al., 2015).

^3^Note: OR, object relative clause; SR, subject relative clause; AN-AN, two animate and singular NPs; IN-AN, one inanimate NP and one animate NP; SG-PL, one singular NP and one plural embedded NP.
