# Supplementary material for: The Peaceful Co-existence of Input Frequency and Structural Intervention Effects on the Comprehension of Complex Sentences in German-Speaking Children
Source: Front Psychol. 2017 Sep 29;8:1590. doi: 10.3389/fpsyg.2017.01590 (PMC5627570; doi:10.3389/fpsyg.2017.01590)
Supplement: Supplementary file 1 [file DataSheet1.pdf]

Supplementary Material

The Interplay of Input Frequency and Structural Intervention in the Comprehension of Complex Sentences in German-Speaking Children

Flavia Adani[1*], Maja Stegenwallner-Schütz[1], Talea Niesel[1]

[1]Department of Linguistics, University of Potsdam, Potsdam, Germany

*** Correspondence:**
Flavia Adani
adani@uni-potsdam.de

1 Stimulus List

P1	Welche Farbe hat der Junge, der kämpft?	practice
P2	Welche Farbe hat der Mann, der den Jungen wäscht?	practice
P3	Welche Farbe hat der Mann, den der Junge wäscht?	practice
P4	Welche Farbe hat der Mann der telefoniert?	practice
1	Welche Farbe hat der Junge, der trinkt?	filler
2	Welche Farbe hat der Mann, der den Jungen trägt?	SR:AN-AN
3	Welche Farbe hat der Mann, der wandert?	filler
4	Welche Farbe hat der Mann, den der Junge kratzt?	OR:AN-AN
5	Welche Farbe hat der Mann, der die Jungen trägt?	SR:SG-PL
6	Welche Farbe hat der Gurt, den der Mann hält?	OR:IN-AN
7	Welche Farbe hat der Mann, der taucht?	filler
8	Welche Farbe hat der Mann, den die Jungen kratzen?	OR:SG-PL
9	Welche Farbe hat der Mann, der badet?	filler

10	Welche Farbe hat der Mann, der schläft?	filler
11	Welche Farbe hat der Mann, der den Jungen hält?	SR:AN-AN
12	Welche Farbe hat der Pulli an der Wäscheleine?	filler
13	Welche Farbe hat der Mann, den die Jungen drücken?	OR:SG-PL
14	Welche Farbe hat der Mann, der trinkt?	filler
15	Welche Farbe hat der Mann, der die Jungen hält?	SR:SG-PL
16	Welche Farbe hat der Baumstamm, den der Mann trägt?	OR:IN-AN
17	Welche Farbe hat der Gurt in der Kiste?	filler
18	Welche Farbe hat der Mann, den der Junge drückt?	OR:AN-AN
19	Welche Farbe hat der Junge, der schläft?	filler
20	Welche Farbe hat der Mann, der den Jungen drückt?	SR:AN-AN
21	Welche Farbe hat der Mann, der schwimmt?	filler
22	Welche Farbe hat der Mann, den der Junge trägt?	OR:AN-AN
23	Welche Farbe hat der Mann, den die Jungen halten?	OR:SG-PL
24	Welche Farbe hat der Schuh im Regal?	filler
25	Welche Farbe hat der Mann, der die Jungen kratzt?	SR:SG-PL
26	Welche Farbe hat der Junge, der liest?	filler
27	Welche Farbe hat der Mann, den die Jungen tragen?	OR:SG-PL
28	Welche Farbe hat der Junge, der wandert?	filler
29	Welche Farbe hat der Mann, der den Jungen kratzt?	SR:AN-AN

30	Welche Farbe hat der Mann, der zaubert?	filler
31	Welche Farbe hat der Schuh, den der Mann drückt?	OR:IN-AN
32	Welche Farbe hat der Baumstamm auf dem Lastwagen?	filler
33	Welche Farbe hat der Pulli, den der Mann kratzt?	OR:IN-AN
34	Welche Farbe hat der Mann, der die Jungen drückt?	SR:SG-PL
35	Welche Farbe hat der Mann, den der Junge hält?	OR:AN-AN
36	Welche Farbe hat der Mann, der liest?	filler

2 Task instructions (and English translation)

Bala (puppet): *Jetzt möchte ich ein neues Spiel mit dir spielen. In diesem Spiel geht es um Farben. Du siehst immer vier Bilder. Auf den Bildern passieren verschiedene Dinge. Du kannst dir jedes Bild genau anschauen. Immer wenn du ein neues Bild sehen kannst, hörst du meine Stimme. Ich beschreibe dir dann, was auf dem Bild passiert. Einige von den Dingen und Menschen von den Bildern haben lauter komische Farben. Manche sind komplett lila andere wieder blau. Dann frage ich dich nach genau einer Farbe auf dem Bild und die sagst du dann. Alles klar? Na dann üben wir das gleich mal.*

English translation: Now, I'd like to play a new game with you. It's a game about colors. You'll always see four pictures. In these pictures, different situations are going on. You should look at each picture very carefully. Each time you see new pictures, you'll hear my voice. I'll describe what is happening in each of the four pictures. Sometimes, the objects and the people in the pictures have strange colors. Some are completely purple, other are blue. Then, I'll ask you the color of one of these things in the pictures. Is everything clear? Let's practice a bit together.

Experimenter: *Hast du verstanden, was BALA dir erklärt hat? Bevor das Spiel jetzt richtig losgeht, zeige ich dir noch ein paar Beispiele zum Üben.*

English translation: Did you understand what Bala has explained? Before the real game starts, I'll show you a couple of examples.

3 Response categories and their frequency of occurrence

Correct: choice of the correct head noun referent, within the correct picture

Reversal error: choice of the correct head noun referent, but within the wrong picture (thematic role reversal)

Agent error: choice of the wrong head noun referent, within the correct picture

Double reversal error: choice of the wrong head noun referent, within the wrong picture

Related distractor error: choice of the correct head noun referent, within the distractor picture

Unrelated distractor error: choice of the wrong head noun referent, within a distractor picture

Figure 3: Percentage of responses, occurring in each response type.

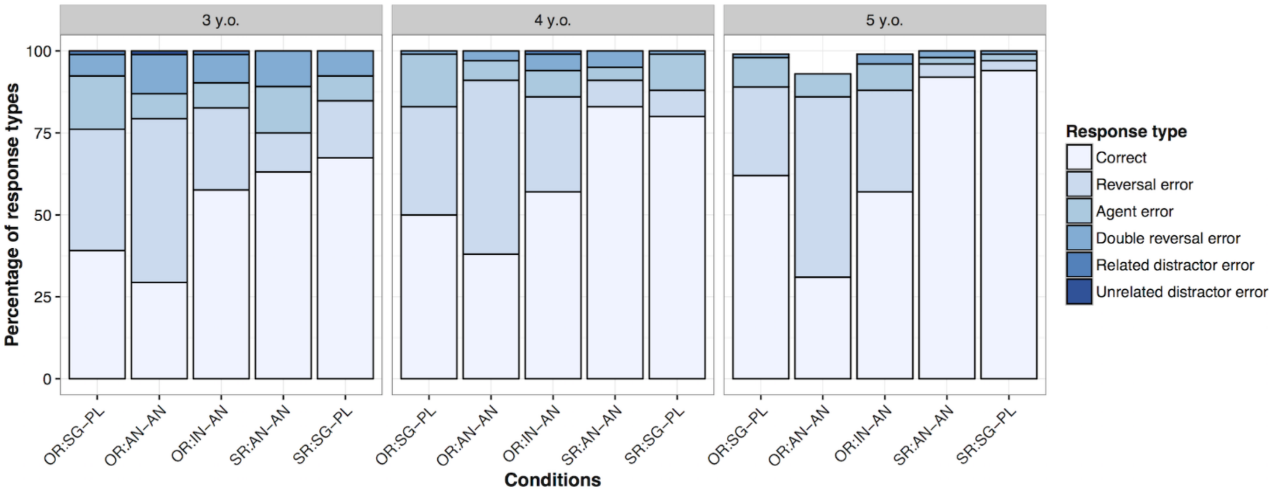

Note: OR: object relative clause; SR: subject relative clause; AN-AN: two animate and singular NPs; IN-AN: one inanimate NP and one animate NP, both singular; SG-PL: one singular NP and one plural NP, both animate.

4 Outputs of the additional models with nested pairwise comparisons

Model for the significant interaction “group x OR:AN-AN vs. OR:SG-PL” (the relevant parameters are highlighted in gray)

Fixed effects	Estimate	SE	z-value	p-value
(Intercept)	0.643	0.134	4.789	<0.001
4 y.o. vs. 3 y.o.	0.637	0.309	2.062	0.039
5 y.o. vs. 4 y.o.	0.532	0.320	1.662	0.096
OR:IN-AN vs. OR:AN-AN	1.277	0.237	5.393	<0.001
SR:AN-AN vs. OR:IN-AN	1.547	0.257	6.013	<0.001
SR:SG-PL vs. SR:AN-AN	0.113	0.262	0.430	0.667
3 y.o.**:** OR:AN-AN vs. OR:SG-PL	-0.566	0.358	-1.581	0.114
4 y.o.**:** OR:AN-AN vs. OR:SG-PL	-0.675	0.340	-1.987	0.047
5 y.o.**:** OR:AN-AN vs. OR:SG-PL	-1.780	0.369	-4.824	<0.001
4 vs. 3 y.o. x OR:IN-AN vs. OR:AN-AN	-0.487	0.572	-0.850	0.395
5 vs. 4 y.o. x OR:IN-AN vs. OR:AN-AN	0.472	0.563	0.838	0.402
4 vs. 3 y.o. x SR:AN-AN vs. OR:IN-AN	1.348	0.546	2.469	0.014
5 vs. 4 y.o. x SR:AN-AN vs. OR:IN-AN	0.968	0.624	1.551	0.121

4 vs. 3 y.o. x SR:SG-PL vs. SR:AN-AN	-0.480	0.530	-0.905	0.365
5 vs. 4 y.o. x SR:SG-PL vs. SR:AN-AN	0.572	0.705	0.811	0.418

Model for the significant interaction group x SR:AN-AN vs. OR:IN-AN (the relevant parameters are highlighted in gray)

Fixed effects	Estimate	SE	z-value	p-value
(Intercept)	0.643	0.134	4.789	<0.001
4 y.o. vs. 3 y.o.	0.637	0.309	2.062	0.039
5 y.o. vs. 4 y.o.	0.532	0.320	1.662	0.096
OR:AN-AN vs. OR:SG-PL	-1.007	0.208	-4.851	<0.001
OR:IN-AN vs. OR:AN-AN	1.277	0.237	5.393	<0.001
SR:SG-PL vs. SR:AN-AN	0.113	0.262	0.430	0.667
3 y.o.: SR:AN-AN vs. OR:IN-AN	0.326	0.367	0.888	0.375
4 y.o.: SR:AN-AN vs. OR:IN-AN	1.674	0.411	4.074	<0.001
5 y.o.: SR:AN-AN vs. OR:IN-AN	2.642	0.500	5.281	<0.001
4 vs. 3 y.o. x OR:AN-AN vs. OR:SG-PL	-0.110	0.492	-0.222	0.824
5 vs. 4 y.o. x OR:AN-AN vs. OR:SG-PL	-1.105	0.497	-2.222	0.026
4 vs. 3 y.o. x OR:IN-AN vs. OR:AN-AN	-0.487	0.572	-0.850	0.395
5 vs. 4 y.o. x OR:IN-AN vs. OR:AN-AN	0.472	0.563	0.838	0.402
4 vs. 3 y.o. x SR:SG-PL vs. SR:AN-AN	-0.480	0.530	-0.905	0.365
5 vs. 4 y.o. x SR:SG-PL vs. SR:AN-AN	0.572	0.705	0.811	0.418
